# *Libifem®* (*Trigonella foenum-graecum*) in conjunction with exercise on muscle strength, power, endurance, and body composition in females aged between 25 and 45 years

**DOI:** 10.3389/fspor.2023.1207013

**Published:** 2023-08-11

**Authors:** Amanda Rao, Paul Clayton, David Briskey

**Affiliations:** ^1^ RDC Clinical, Brisbane, QLD, Australia; ^2^ Department of Personalized and Preventive Medicine, Institute of Interdisciplinary Medicine, Moscow, Russia

**Keywords:** muscle strength, female exercise, body composition, fenugreek, resistance training

## Abstract

**Introduction:**

This study examined the effects of *Libifem*® on exercise performance and body composition in females 25–45 years old.

**Methods:**

Participants were randomized to three equal groups to consume: 600 mg *Libifem*®/day, 300 mg *Libifem*®/day or a placebo for 8 weeks. Participants completed a whole-body exercise program three times a week for 8 weeks. At baseline, week 4 and week 8, muscle strength and endurance, functional threshold power, body composition, and sex hormones were measured. At week 8, all three groups increased leg press 1RM compared to baseline.

**Results:**

A significant difference between group treatment effect was seen for leg press at week 8 (*p* = 0.045), with the 600 mg *Libifem*® group significantly increasing their leg press 1RM compared to placebo (*p* = 0.014). The 600 mg *Libifem*® group significantly reduced their total fat mass (0.96 kg loss) from baseline compared to placebo group (0.09 kg gain). There was no significant difference in fat mass for the 300 mg *Libifem*® group (0.23 kg loss). The 600 mg *Libifem*® group had a significant increase in lean mass compared to both the 300 mg and placebo groups (*p* = 0.011 and 0.009, respectively).

**Discussion:**

Overall, there were significant and dose-related changes in body composition and ergogenic parameters, comparable with previous findings in males.

**Clinical Trial Registration:**

This trial was registered with the Australian and New Zealand Clinical Trials registry [ACTRN12618001538235].

## Introduction

Regular physical activity is associated with improvements in body composition, bone, and cardiovascular health (1), as well as positively affecting mood and mental health (2). A strong interest in performance enhancement via natural and legal (and safe) routes exists, and while much of this research has focused on male performance, a growing demand for analogous research applicable to women arises. Phytochemical research is currently investigating herbal strategies designed to specifically improve training, recovery, and performance among female athletes and competitors (3).

Many of the benefits associated with physical activity are largely influenced by the sex hormones. Physical activity, in particular resistance training, is impacted by the presence of circulating testosterone in both males and females. Resistance training is well known to induce muscle hypertrophy, increase strength (as measured by 1RM) (4–8), increase lean muscle mass (9, 10), and help reduce fat mass by increasing resting metabolic rate (5).

Testosterone stimulates protein synthesis and inhibits protein degradation within muscles, leading to promotion of muscle growth and increases in muscle strength (11), whereas oestrogen can exert a broadly analogous effect by increasing the anabolic response to exercise in females (12).

The herb *Trigonella foenum-graecum*, known as fenugreek, belongs to the Fabaceae family. Traditionally, fenugreek has been used as a food, condiment, spice, traditional medicine, and health supplement (12, 13). Based on its active constituents, including the amino acid 4-hydroxyisoleucine, saponins, and numerous other phytochemicals with varying biological and pharmacological activities (13–15), fenugreek has been shown to exert positive effects in diabetes, inflammation, and some types of cancer (15).

Studies using fenugreek, specifically in females, have mainly focused on the amelioration of menopausal symptoms. *Libifem®*, an extract of fenugreek, standardised to 50% of furostanol saponins, has been shown to bind to E2 receptors and induce expression of E2-responsive genes (16) and improve sexual function in both pre- and postmenopausal females (17, 18). Fenugreek has been shown to increase free oestrogen and testosterone levels in females (17, 18) and testosterone in males (19, 20), via re-partitioning mechanisms including the displacement of testosterone from relatively low-affinity binding sites on, i.e., serum albumen (21). These pharmacological effects would be expected to improve aspects of muscle function. A previous study on females undertaking resistance training with fenugreek supplementation (22) showed that completing either two or three resistance training sessions per week showed similar increases in muscle strength and lean soft tissue mass, with those in the higher-frequency group showing improvements in body mass. However, the full effects of fenugreek on females and exercise performance have not yet been studied.

The aim of this study was to examine changes in muscle strength and endurance, as well as body composition, leg power, muscle recovery, pathological markers, and quality of life in response to an 8-week bodyweight resistance training program in females aged 25–45 years, with varying doses of *Libifem®* or a placebo. It was hypothesised that participants on *Libifem®* would show an increase in muscle strength, power, and endurance, which would, in turn, positively impact body composition at a faster rate than a placebo. It was also hypothesised that participants on the higher dose of *Libifem®* would elicit greater improvements in performance compared with both the placebo and low-dose *Libifem®* group.

## Methods

This study was a double-blind, randomised, placebo-controlled, multi-site (Brisbane and Gold Coast, Australia) interventional study conducted over an 8-week treatment duration, utilising two active and one placebo group. It was approved by Bellberry Human Research Ethics Committee (HREC 2016-04-307) and registered on the Australian and New Zealand Clinical Trials Registry (ACTRN12616000938404).

The participants were recruited from databases and public media outlets, and following preliminary screening via telehealth consult, potential participants attended the clinic for an information session before providing their written consent for inclusion into the study. Consenting participants underwent a health assessment that included lifestyle, current medications, physical assessment, and medical history. Muscle strength, power, endurance, body composition, and quality of life were also assessed upon enrolment. Within the week pre-treatment, the participant's blood was collected for baseline analysis. Once all baseline measures were successfully completed, the participants were enrolled in the trial and randomly allocated to the placebo comparator group or one of the two active intervention groups (300 mg of *Libifem®*/day or 600 mg of *Libifem®*/day) using Random Allocation Software (www.sealedenvelope.com). This study was conducted in a double-blind manner such that both the investigators and participants were blind to treatment allocation.

Inclusion criteria included females aged 25–45 years with a body mass index (BMI) of 18.5–29.9 kg/m^2^. The participants had to be undertaking low-impact cardiovascular exercise including but not limited to cycling, swimming, or walking at least once a week, but no more than five times per week, but not undertaking any resistance training exercises. The participants had to be able to provide written informed consent and willing to participate in an exercise program three times per week for 8 weeks. They were excluded based on the following criteria: currently undertaking resistance training exercise; consumed any dietary supplements within the previous 3 months; had known hypersensitivity to herbal drugs, nutritional supplements, or foods; or completed any other clinical trial within 6 months prior to their enrolment. Other exclusion criteria consisted of clinically significant medical conditions including, but not limited to, cardiovascular, neurological, psychiatric, renal, immunological, endocrine (such as uncontrolled diabetes or thyroid disease), or haematological abnormalities that were uncontrolled; prolonged (≥6 weeks) medication with corticosteroids, antidepressants, anticholinergics, or any other drugs that may have had an influence on the outcome of the study; severe pulmonary dysfunction (uncontrolled bronchial asthma and/or chronic obstruction); history of orthopaedic injuries or surgeries in the previous 6 months; active smokers; substantial alcohol consumption (≥21 drinks per week); drug use; or females that were pregnant or lactating, including those actively trying to fall pregnant.

Primary measurement outcomes included a one-repetition maximum (1RM) leg press and bench press. For 1RM testing, participants stretched major muscle groups of the lower limbs followed by 10 repetitions of leg press at 50% of estimated 1RM and a 2-min rest. The weight on the leg press was then increased to approximately 70% of 1RM, and the participants completed four to six repetitions followed by a 2-min rest. Weight was added onto the leg press to approximately 90% of estimated 1RM, and the participants completed one repetition followed by a 2-min rest. Weight was then increased to 100% of estimated 1RM, and the participants attempted to complete a repetition. If successful, the weight was increased, and if unsuccessful, the weight was decreased. The participants were given up to seven attempts to achieve 1RM. They were rested for 3 min in between attempts.

Secondary measurement outcomes included 80% of 1RM leg press and bench press repetitions to fatigue. This was measured following a 5-min rest after completing the 1RM testing, where the participants completed one set of 80% of 1RM leg press for as many reps as possible. The participants again rested for further 5 min before an identical protocol was followed for bench press. Other secondary measures included body composition [lean muscle mass, fat mass, body fat percentage, BMI, total body mass, android/gynoid ratio, and visceral adipose tissue (VAT) mass] measured by dual-energy x-ray absorptiometry (DEXA); muscle recovery [creatine kinase (CK), lactate dehydrogenase (LDH)]; pathology [stress indicators (cortisol), liver and kidney safety markers, testosterone, E/LFT, C-reactive protein, homocysteine, blood glucose, and full blood count]; evaluation of dose, safety, and tolerability of *Libifem*®; an assessment of quality of life; and leg power as measured by a 20-min Functional Threshold Power (FTP) test. The protocol used for FTP testing was based on the existing validated protocols (23, 24). Following a 15-min warm-up, the participants completed a 20-min FTP test on a bicycle attached to a Wahoo KICKR® Power Trainer (Wahoo Fitness) and wore a heart rate monitor (Polar Electro Inc.). Each participant was instructed to perform the highest possible mean power output for the duration of the test. Standardised verbal encouragement was provided to each participant, and water could be consumed *ad libitum*. FTP was determined as the mean power output over the test duration.

Within a week from baseline testing, the participant's fasted (10 h) blood was collected at an accredited local pathology laboratory (Queensland Medical Laboratory) for pathology analysis [stress indicators (cortisol), liver and kidney safety markers, testosterone, E/LFT, C-reactive protein, homocysteine, blood glucose, and full blood count]. Blood samples for markers of exercise recovery (CK and LDH) were collected the following day (approximately 24 h) after the exercise test. This process was repeated at the completion of the study (week 8). All blood draws were collected in the morning, at the same time for each participant to ensure any variation due to diurnal rhythm was minimised. All blood was drawn into EDTA or serum vacutainers from a vein in the antecubital fossa. Once collected, the EDTA sample was immediately centrifuged, while the serum sample was allowed to clot for at least 30 min prior to centrifugation. Both samples were centrifuged at 1,400 × g for 10 min at 4°C. Once spun, the blood was immediately analysed or stored at −80°C until analysis.

**Figure 1:**
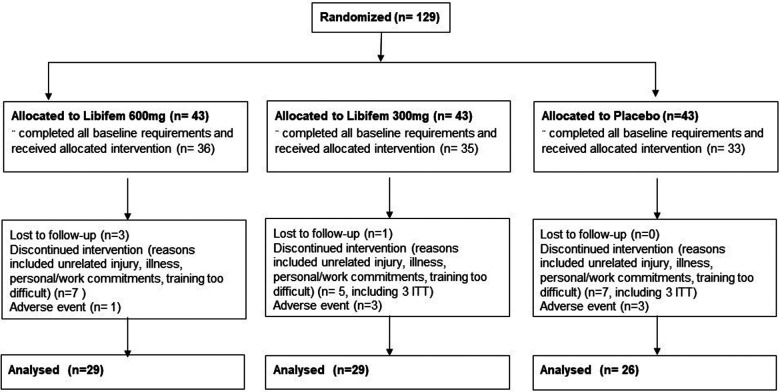
Participant flow diagram with designated allocations, participant withdrawal, and analysis.

One hundred and twenty-eight participants were enrolled and equally randomised across three treatment arms that included 600 mg of *Libifem®*/day (*n *= 29), 300 mg of *Libifem®*/day (*n *= 29), and a placebo group (*n *= 26) (Figure 1). The active capsules contained a standardised *Trigonella foenum-graecum* seed extract, *Libifem*®, supplied by Gencor Pacific Ltd., and the placebo product contained maltodextrin. Both treatments consisted of dose matched capsules divided into two daily doses, once with a morning meal and once with an evening meal.

The participants were asked to take the allocated product and complete a resistance training program consisting of both upper and lower body exercises focusing on all major muscle groups. They completed three training sessions per week during weeks 1–3 and 5–7 and two sessions during assessment weeks 4 and 8. Testing sessions during weeks 4 and 8 constituted their third weekly session.

At the mid-point (week 4), the participants were assessed on resting heart rate (HR) and blood pressure (BP); anthropometric measurements including height, weight, waist, and hip circumference; 1RM leg press and bench press; and 80% of 1RM bench leg press and bench press repetitions to fatigue.

Upon completion of the study (week 8), an assessment identical to baseline was completed. At both the mid-point and endpoint of the study (weeks 4 and 8), the participants were asked to provide details regarding any lifestyle changes (diet, exercise, medication) and any adverse effects. It should be noted that the phase of each participant's menstrual cycle at the time of testing was not recorded for this study.

Statistical analyses were conducted using SPSS software. All data were initially assessed for normality, and statistical differences were assessed by ANOVA and RMANOVA for change over time and treatment. Post hoc analyses were undertaken to assess the differences between treatment groups. Significance was assumed when *p* < 0.05. A sample size of 26 per group was calculated based on the power to detect a 5% change in 1RM leg/bench press (effect size of 0.56, alpha error probability of 0.05, power of 0.95) (25).

## Results

One hundred and twenty-nine participants were recruited and enrolled into the study, with 74 completing the full requirements. Of the 54 participants that did not complete the study, 26 did not complete the baseline requirements, 7 dropped out due to adverse events, 5 were lost to follow-up, 5 withdrew due to personal reasons, and 12 withdrawals were due to COVID-19 restrictions. Of the participants that did not complete the study, 10 provided data for at least the week 4 time-point, and these were included in modified intention to treat analysis that included all randomised participants who had at least one post-baseline measurement for the primary outcome. Data were therefore analysed for 84 participants (Figure 1).

No significant differences between the active treatment and placebo groups at baseline for age, anthropometric measures, pathology, lifestyle factors, quality of life, or exercise measurements were noted (Tables 1–3). No significant differences between groups for any biochemistry safety parameter measured were found. All biochemical safety markers were within the normal range at baseline and remained stable and within normal reference ranges at week 8 (Table 3). No significant differences between groups for exercise session compliance were reported, with compliance of approximately 82% for all three trial groups. All data were normally distributed.

**Table 1:** Demographics of study population.

	600 mg of *Libifem®* (*n *= 29)	300 mg of *Libifem®* (*n* = 29)	Placebo (*n* = 26)
Age	34.75 (4.79)	34.69 (5.84)	33.73 (5.22)
Weight (kg)	62.44 (7.78)	64.23 (10.07)	64.84 (5.68)
Height (cm)	165.11 (7.15)	166.78 (6.09)	167.13 (7.21)
BMI (kg/m^2^)	22.94 (0.62)	23.08 (0.79)	23.29 (0.86)

Data are shown as mean (SD).

**Table 2:** Anthropometric measurements.

	600 mg of *Libifem®* (*n* = 29)			300 mg of *Libifem®* (*n* = 29)			Placebo (*n* = 26)		
	Baseline	Week 4	Week 8	Baseline	Week 4	Week 8	Baseline	Week 4	Week 8
Waist circumference (cm)	75.5 (6.4)	75.4 (6.5)	75.2 (6.9)	76.8 (6.4)	77.9 (7.0)	76.8 (7.0)	78.5 (6.9)	78.7 (7.7)	78.3 (7.5)
Hip circumference (cm)	99.2 (5.9)	97.6 (6.7)	97.5 (6.9)	99.2 (7.8)	100.1 (7.4)	99.2 (8.2)	99.4 (5.4)	97.9 (4.2)	99.1 (4.7)
Blood pressure—systolic (mmHg)	114.1 (15.6)	118.1 (13.2)	114.8 (9.9)	113.8 (12.2)	115.2 (10.9)	112.6 (11.6)	119.7 (12.0)	120.4 (13.5)	120.1 (14.6)
Blood pressure—diastolic (mmHg)	77.2 (11.8)	76.3 (9.1)	75.0 (9.0)	74.1 (9.4)	72.6 (9.1)	71.8 (9.1)	78.0 (8.6)	78.6 (11.4)	78.0 (8.9)

Data are shown as mean (SD).

**Table 3:** Biochemical blood safety markers at baseline of the study population and the change following 8 weeks of supplementation and training.

	Baseline			Change from baseline		
	600 mg of Testofen (*n *= 29)	300 mg of Testofen (*n *= 29)	Placebo (*n *= 26)	600 mg of Testofen (*n *= 29)	300 mg of Testofen (*n *= 29)	Placebo (*n *= 26)
Oestrogen (pg/ml)	294.69 (318.97)	322.88 (369.5)	333 (285.24)	6.42 (356.95)	−131.56 (357.1)	97.59 (371.35)
Progesterone (ng/ml)	6.88 (12.38)	9.12 (14.0)	8.36 (12.4)	−1.00 (7.95)	−2.4 (19.07)	−1.91 (16.9)
Testosterone (nmol/L)	0.93 (0.28)	0.87 (0.21)	0.92 (0.35)	0.03 (0.31)	−0.02 (0.32)	0.04 (0.27)
SHBG (nmol/L)	114.73 (64.54)	89.88 (78.32)	78.95 (42.25)	−1.15 (20.68)	4.24 (25.66)	2.27 (22.7)
Insulin (mU/L)	5.77 (2.67)	4.88 (2.33)	5.41 (2.06)	−0.27 (2.65)	1.52 (4.02)	1.09 (2.74)
Sodium (mmol/L)	139.88 (2.21)	139.72 (2.61)	138.5 (2.41)	−0.39 (2.51)	−0.56 (2.02)	0.82 (3.14)
Potassium (mmol/L)	4.41 (0.377)	4.26 (0.34)	4.34 (0.38)	−0.1 ± 0.3	−0.04 (0.38)	0.05 (0.44)
Chloride (mmol/L)	105.81 (2.32)	105.64 (2.74)	105.18 (1.97)	0.12 (2.07)	−0.12 (2.22)	0.64 (2.84)
Bicarbonate (mmol/L)	27.04 (1.82)	26.92 (2.06)	25.86 (2.66)	−0.04 (1.97)	0.16 (1.84)	0.55 (2.58)
Anion gap (mmol/L)	11.46 ± 1.5	11.4 (1.85)	11.72 (2.41)	−0.69 (2.51)	−0.84 (2.44)	−0.32 (3.08)
Glucose (mmol/L)	4.72 (0.43)	4.72 (0.32)	4.57 (0.32)	−0.01 (0.38)	−0.06 (0.4)	0.15 (0.33)
Urea (mmol/L)	4.47 (1.31)	5.31 (2.35)	5.05 (1.53)	−0.09 (0.89)	−0.31 (1.59)	−0.3 (0.97)
Creatine (μmol/L)	67.04 (8.92)	69.72 (9.9)	66.45 (11.32)	0.15 (7.25)	−0.56 (7.3)	−1.36 (6.79)
eGFR (ml/min)	82.8 (5.07)	78.17 (7.41)	75.5 (11.36)	N/A	N/A	N/A
Urate (mmol/L)	0.27 (0.050)	0.3 (0.05)	0.27 (0.05)	−0.01 (0.03)	0.00 (0.03)	−0.01 (0.05)
Total bilirubin (μmol/L)	11.88 (6.45)	12.08 (4.65)	11.68 (5.2)	−0.12 (4.79)	−0.2 (4.85)	−0.68 (4.54)
Alkaline phosphatase (U/L)	57 (15.92)	55.12 (17.41)	55.55 (14.54)	2.04 (6.16)	−1.4 (8.3)	2.73 (13.58)
GGT (U/L)	16.27 (10.54)	17.12 (13.45)	17.41 (20.53)	−1.18 (3.51)	−2.12 (11.25)	3.59 (11.68)
ALT (U/L)	18.77 (15.05)	19.8 (9.29)	20.41 (20.36)	−0.31 (13.04)	−2.56 (7.98)	−0.9 (22.05)
AST (U/L)	23.77 (16.61)	23.28 (5.3)	31.86 (47.4)	−1.48 (15.03)	−2.08 (4.51)	−8.71 (49.84)
Lactate dehydrogenase (U/L)	154.62 (29.02)	163.04 (36.05)	171.36 (39.63)	0.65 (15.43)	−4.64 (18.26)	−3.91 (48.94)
Calcium (mmol/L)	2.32 (0.07)	2.36 (0.1)	2.32 (0.09)	−0.01 (0.08)	−0.10 (0.50)	0.04 (0.11)
Corrected calcium (mmol/L)	2.35 (0.08)	2.36 (0.09)	2.35 (0.08)	0.04 (0.2)	0.001 (0.07)	0.03 (0.08)
Phosphate (mmol/L)	1.13 (0.19)	1.22 (0.10)	1.19 (0.13)	0.04 (0.16)	0.003 (0.19)	−0.05 (0.24)
Protein (g/L)	67.73 (3.5)	68.76 (2.99)	68.81 (4.43)	−0.77 (2.89)	−0.16 (2.97)	1.09 (3.57)
Albumin (g/L)	41.27 (3.14)	42.52 (2.4)	41.5 (3.51)	−0.27 (2.32)	−0.64 (2.04)	0.05 (3.08)
Globulin (g/L)	26.46 (3.15)	26.24 (2.68)	27.32 (3.17)	−0.5 (2.16)	0.48 (1.96)	0.82 (1.53)
Cholesterol (mmol/L)	4.58 (0.57)	4.82 (0.72)	4.77 (0.76)	0.06 (0.41)	−0.08 (0.51)	−0.08 (0.57)
Triglycerides (mmol/L)	0.78 (0.31)	0.93 (0.36)	0.78 (0.4)	−0.05 (0.23)	−0.1 (0.35)	−0.04 (0.37)
CK (U/L)	144.69 (202.33)	126.96 (72.56)	346.5 (1,107.82)	−43.19 (192.11)	−25.72 (83.13)	−159.23 (1,175.74)
CRP (mg/L)	0.73 (2.11)	1.04 (3.08)	1.9 (4.42)	0.15 (2.66)	1.5 (4.67)	0 (3.73)
Homocysteine (μmol/L)	10.97 (3.17)	11.51 (2.9)	10.5 (3.2)	0.57 (4.83)	−0.88 (3.71)	−0.63 (1.96)
Cortisol (nmol/L)	447.69 (172.29)	417.6 (186.67)	404.09 (168.68)	23.85 (138.28)	−8.52 (159.34)	1.36 (150.91)

Data are shown as mean (SD).

SHBG, sex hormone-binding globulin; eGFR, estimated glomerular filtration rate; GGT, gamma-glutamyl transferase; ALT, alanine transaminase; AST, aspartate aminotransferase; CK, creatine kinase; CRP, C-reactive protein; N/A, not applicable.

**Figure 2:**
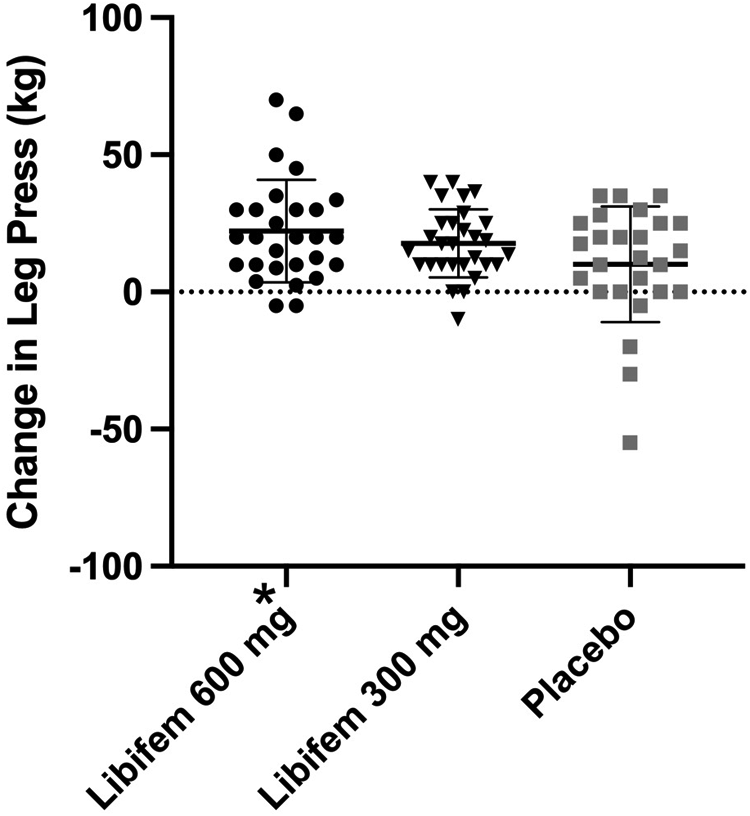
Change in 1RM leg press (kg) following 8 weeks of resistance training and supplementation with either 600 mg of *Libifem®*, 300 mg of *Libifem®*, or placebo.

A significant difference of between-group treatment effect was seen for leg press at week 8 [F (2, 82) = 0.122, *p* = 0.045]. All three groups improved their 1RM leg press from baseline to week 8 (22.17 kg, 17.68 kg, and 10.12 kg for the 600 mg, 300 mg, and placebo groups, respectively). The 600 mg *Libifem®* group significantly improved (*p* = 0.014) from baseline compared with the placebo at week 8 (Table 4 and Figure 2).

**Table 4:** Exercise testing.

	600 mg of *Libifem®* (*n* = 29)			300 mg of *Libifem®* (*n* = 29)			Placebo (*n* = 26)		
	Baseline	Week 4	Week 8	Baseline	Week 4	Week 8	Baseline	Week 4	Week 8
Maximum bench press (kg)	34.76 (7.87)	38.25^^^ (8.91)	39.88^^^ (8.71)	37.91 (10.28)	41.75^^^ (10.64)	43.9^^^ (11.54)	36.88 (8.35)	42.68^^^ (9.13)	43.04^^^ (9.14)
80% bench press repetitions (*n*)	8.1 (3.24)	7.82 (3.57)	7.52 (3.64)	7.9 (3.03)	7.08 (3.21)	5.52 (2.47)	8.12 (2.89)	6.72 (2.75)	8.08 (7.27)
Maximum leg press (kg)^#^	167.76 (29.57)	182.3^^^ (35.68)	190.78^^^* (34.19)	170.51 (38.70)	189.23^^^ (35.46)	188.19^^^ (36.18)	183.15 (29.84)	195.99^^^ (32.56)	193.27 (29.37)
80% leg press repetitions (*n*)	19.45 (7.56)	22.81 (7.88)	18.33 (8.23)	19.17 (12.64)	19.12 (11.44)	16.62 (10.57)	19.35 (10.26)	21.68 (9.98)	17.92 (9.32)
Functional threshold power (Watts)	121.93 (32.11)		128.48 (36.89)	120.66 (35.32)		128 (38.55)	127.42 (29.79)		134.52 (22.16)
Functional threshold power heart rate (bpm)	155.18 (18.65)		152.33 (16.10)	151.07 (16.19)		151.54 (17.77)	157.69 (12.69)		160.4 (14.05)
Resting heart rate (bpm)	70.31 (12.07)	65.18 (16.60)	65.72 (10.56)	67.07 (10.52)	68.92 (10.66)	67.52 (10.46)	64.19 (7.53)	72.12 (10.91)	67.92 (10.15)

Data are shown as mean (SD).

^#^ Significant between-group statistical difference ANOVA.

^ Significant within group (change from baseline).

* Significant difference for change from baseline between 600 mg and placebo.

Significant increases (*p *< 0.05) from baseline in 1RM bench press were observed for all three groups (5.12 kg 600 mg *Libifem®*, 5.98 kg 300 mg *Libifem®*, 6.15 kg placebo); however, no significant differences between the groups were found. No differences were observed over time or between groups for the 80% max bench or leg press repetitions.

A significant between-group treatment effect was seen for the change in total fat mass at week 8 [F (2, 70) = 3.90, *p *= 0.025] (Table 5). The 600 mg *Libifem®* group had a significant decrease from baseline total fat mass compared with placebo, losing on average 0.96 kg over the 8 weeks, compared with a 0.23 kg loss in the 300 mg group and a 0.09 kg gain in the placebo group.

**Table 5:** Body composition.

	600 mg of *Libifem®* (*n* = 29)		300 mg of *Libifem®* (*n* = 29)		Placebo (*n* = 26)	
	Baseline	Week 8	Baseline	Week 8	Baseline	Week 8
Total mass (kg)	62.12 (7.89)	62.32 (7.98)	65.09 (10.49)	65.01 (10.99)	66.29 (6.27)	66.46 (6.24)
Total android mass (kg)	4.11 (0.79)	4.10 (0.78)	4.26 (1.01)	4.27 (0.97)	4.53 (0.81)	4.57 (0.79)
Total gynoid mass (kg)	10.80 (1.49)	10.80 (1.55)	11.31 (1.91)	11.32 (1.99)	11.51 (1.40)	11.45 (1.30)
Android/gynoid ratio	0.74 (0.17)	0.71 (0.18)	0.73 (0.13)	0.72 (0.12)	0.78 (0.18)	0.78 (0.20)
VAT mass (g)	211.68 (112.01)	175.23 (105.98)	170.53 (111.83)	166.16 (85.18)	221.14 (108.52)	235.76 (116.06)
Fat mass trunk (kg)^#^	6.77 (2.98)	6.20 (2.82)*	7.24 (3.21)	7.10 (2.77)	7.68 (2.58)	7.75 (2.77)
Fat mass total (kg)	17.49 (5.40)	16.58 (5.41)*	18.01 (6.44)	17.79 (5.86)	18.69 (4.60)	18.78 (4.75)
Lean trunk mass (kg)	21.21 (2.78)	21.83 (2.91)	22.59 (2.97)	22.70 (3.38)	23.06 (2.73)	23.16 (2.75)
Lean leg mass (kg)^#^	14.11 (1.66)	14.38 (1.85)*^,^**	14.65 (2.00)	14.68 (2.28)	14.68 (1.52)	14.63 (1.62)
Lean total (kg)^#^	42.42 (4.74)	43.47 (5.28)*	44.68 (5.33)	44.83 (6.30)	45.25 (4.49)	45.33 (4.69)

Data are shown as mean (SD).

^#^ Significant between-group statistical difference ANOVA.

* Significant difference for change from baseline between 600 mg and placebo groups.

** Significant difference for change from baseline between 600 mg and 300 mg groups.

**Figure 3:**
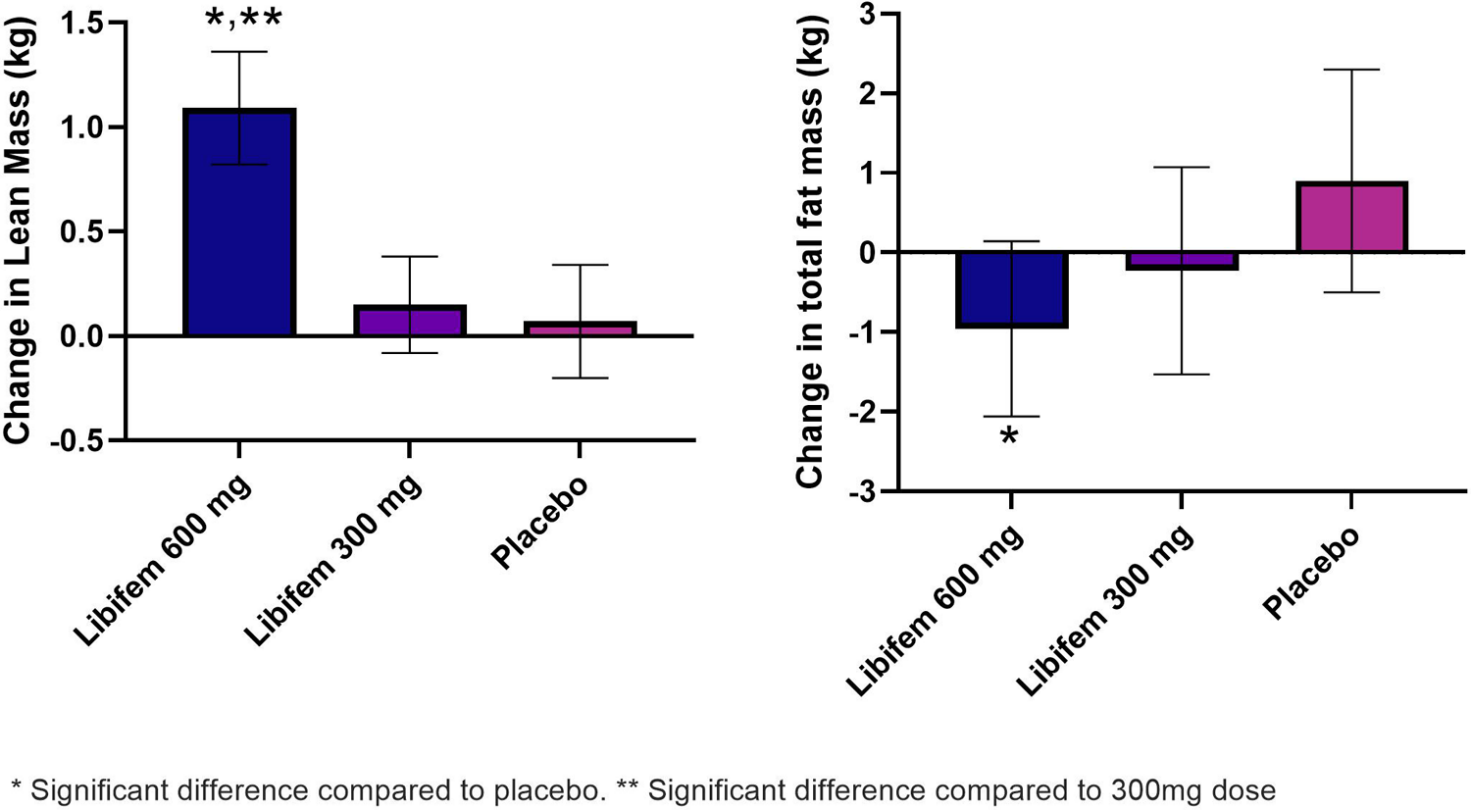
Change in lean mass (kg) and total fat mass (g) as measured by DEXA following 8 weeks of resistance training and supplementation with either 600 mg of *Libifem®*, 300 mg of *Libifem®*, or placebo.

A significant between-group treatment effect was observed for the change in trunk mass fat at week 8 [F (2, 70) = 3.45, *p* = 0.037] (Table 5). The 600 mg *Libifem®* group had a significant decrease from baseline trunk fat mass compared with placebo, losing on average 0.59 kg over the 8 weeks, compared with 0.14 kg loss in the 300 mg group and a 0.07 kg gain in the placebo group (Figure 3).

A significant between-group treatment effect was reported for the change in total lean mass at week 8 [F (2, 70) = 4.73, *p* = 0.012] (Table 5). The 600 mg *Libifem®* group had a significant increase in lean mass compared with both the 300 mg and placebo groups (*p *= 0.011 and 0.009, respectively), gaining 1.09 kg compared with 0.152 kg and 0.079 kg in the 300 mg and placebo groups, respectively, over 8 weeks (Figure 3).

Complementing an increase in total lean mass, the 600 mg group had a significant increase in total lean mass in the legs (280 g) compared with a reduction in the placebo group (−70 g) at week 8 (*p* = 0.022).

No other significant changes in body composition were determined.

One serious adverse event, a bowel obstruction, was reported, which was not related to the trial product (placebo). The participant has since fully recovered. Six other adverse events occurred, and each participant withdrawn from the study. Reasons for dropout in the placebo group (*n* = 1) included joint pain and tingling and, in the active groups (*n* = 5), bloating, knee pain, acne breakout, and reflux (two participants).

## Discussion

This study examined the effect of *Libifem®* on body composition and muscle strength, power, endurance, and recovery in females undertaking resistance training. Daily supplementation with 600 mg dose of *Libifem®* in conjunction with resistance training significantly increased 1RM values and lean mass compared with resistance training alone (placebo) in females. The 600 mg *Libifem®* group decreased total fat mass (−0.96 kg) and trunk fat mass (−0.59 kg), increased lean mass (+1.09 kg), and improved 1RM leg press compared with the placebo group. In both the 300 mg and 600 mg *Libifem®* groups, significant intra-group changes for total fat mass, trunk fat mass, and lean mass from baseline to week 8 that did not occur in the placebo group were identified.

Supporting the findings of this study, a study conducted by Poole and colleagues (2010) on 49 resistance trained men found that supplementation with 500 mg of fenugreek for 8 weeks resulted in reduced body fat percentage and increased 1RM leg press and bench press (26). Other studies conducted by Rao et al. (25) and Wankhede et al. (27) used a similar protocol to Poole and the current study, with a dose of 300 mg of fenugreek twice daily finding that fenugreek improved 1RM leg press and body fat percentage (27). Although the present study focuses on females, the results of fenugreek on resistance training and body composition appear transferable between genders, with similar changes being seen across studies.

A study by Taylor and colleagues (2011) combining the fenugreek extract with creatine found that the combination supplement increased 1RM bench press and leg press and significantly increased lean mass. However, some of these changes were also seen in the placebo group and creatine/dextrose group (28). While the study by Taylor and colleagues was also conducted on resistance-trained males and had an additional component in the treatment group, similar improvements were seen to the current study. Based on this evidence, it can be assumed that fenugreek can significantly improve factors associated with both males and females who are resistance trained or not.

Following 8 weeks of fenugreek supplementation, no significant changes in testosterone levels in any group were seen, with all values remaining within the normal range. This is consistent with other studies of resistance training that have shown increases in muscle mass but no increase in testosterone or free testosterone levels (9, 10). The lack of change in testosterone could be due to the timing of the blood sampling. Other studies have indicated that testosterone and free testosterone trainings are only temporarily raised during exercise once the participants have reached exhaustion, with levels returning to baseline within 24 h (29). Therefore, it is possible that change in testosterone due to the exercise could have been missed. There could also be a gender differentiation with respect to fenugreek's effects on testosterone. An identical study of the *Trigonella foenum-graecum* extract in males similarly showed positive effects on 1RM values and body composition, with significant increases in testosterone in the 600 mg group (19). Males self-evidently produce more testosterone than females, especially during resistance training (30). This is thought to be due to adaptive changes in the synthesis of testosterone and/or secretory capacity of Leydig cells, adrenergic stimulation, plasma volume reductions, and lactate-stimulated secretion (30). It is unlikely that the differences are due to testosterone in females being used in the conversion to oestradiol, as oestradiol circulates in picomolar concentrations compared with nanomolar concentrations of testosterone (31). It could be dependent upon the stage of the menstrual cycle, as testosterone significantly fluctuates throughout the cycle (32). The amount of free circulating testosterone is determined by testosterone production, which, when compared with males, is significantly less in females (31). A previous research has shown that *Libifem®* can increase levels of free oestradiol (17) and may help explain the resulting enhanced anabolic response to exercise (12). Future research using *Libifem®* in females may benefit by having a closer focus on oestradiol rather than testosterone.

Exercise studies in females present a number of challenges not typically encountered in male-only studies. The most confounding factor in female-only studies is the potential for hormone shifts linked to the menstrual cycle and menopause. Hormonal changes throughout a study period have the potential to influence cardiovascular and respiratory systems, thermoregulation, and injury/repair mechanisms (33–35), all of which can impact exercise outcomes. A limitation of this study was the lack of evaluation of the participant's menstrual cycles and the effect hormone fluctuation may have had on the results. However, as menstrual cycles were not monitored in this study, further testing is required on this. Testing of the hormone levels or monitoring the menstrual cycle phases may have assisted in evaluating the true effects of *Libifem®* and how testosterone levels may have improved or affected the measured outcomes. Further research including variables to account for these changes in hormones may be beneficial to assist in the understanding of this phenomenon.

Overall, this study was able to find that supplementation with *Libifem®* improved 1RM measurements and body composition. The major outcomes from this study can be applied to women's resistance training, as there is a need for natural products that benefit body composition. Despite the potential variables involved in female-only exercise studies, the beneficial effects of *Libifem®* following resistance training were evident, and the product was well tolerated. Therefore, this study has the potential to increase the exercising capabilities and alter the physique of females partaking in resistance-based exercise—an area of increasing popularity, but typically under targeted by both science and commercial products.

The developing science of phyto- and nutrimodulation of multiple metabolic sequences using natural and often food-based products opens a new chapter in sporting enhancement. The enhancement effects of any one product are generally slight, but the safety of these compounds permits concomitant use. Given the different mechanisms of action of natural and food-based products (36–40), it is possible that multiple supplements may have an additive effect offering greater advantages to those who know the science. The effect that combining multiple supplements together may have is an area of increasing interest and a potential focus for future studies.

## Data Availability

The original contributions presented in the study are included in the article, and further inquiries can be directed to the corresponding author.
